# Deletion of interleukin-6 alleviated interstitial fibrosis in streptozotocin-induced diabetic cardiomyopathy of mice through affecting TGFβ1 and miR-29 pathways

**DOI:** 10.1038/srep23010

**Published:** 2016-03-14

**Authors:** Yang Zhang, Jing-Hao Wang, Yi-Yuan Zhang, Ying-Zhe Wang, Jin Wang, Yue Zhao, Xue-Xin Jin, Gen-Long Xue, Peng-Hui Li, Yi-Lin Sun, Qi-He Huang, Xiao-Tong Song, Zhi-Ren Zhang, Xu Gao, Bao-Feng Yang, Zhi-Min Du, Zhen-Wei Pan

**Affiliations:** 1 Department of Pharmacology (Key Laboratory of Cardiovascular Medicine Research, Ministry of Education; State-Province Key Laboratories of Biomedicine-Pharmaceutics of China), Harbin Medical University, Harbin, Heilongjiang 150081, P. R. China; 2 Institute of Clinical Pharmacology, The 2nd Affiliated Hospital of Harbin Medical University, Xuefu Road, Harbin, Heilongjiang, China; 3 Department of Biochemistry and Molecular Biology, Harbin Medical University, Harbin, Heilongjiang 150081, P. R. China; 4 Department of Cardiology, The 3rd affiliated hospital of Harbin Medical University Cancer Hospital, Institute of Metabolic Disease, Heilongjiang Academy of Medical Science, Harbin, 150081, P. R China; 5 Department of Pharmacology and Therapeutics, Melbourne School of Biomedical Sciences, Faculty of Medicine, Dentistry and Health Sciences, The University of Melbourne

## Abstract

Interleukin 6 (IL-6) has been shown to be an important regulator of cardiac interstitial fibrosis. In this study, we explored the role of interleukin-6 in the development of diabetic cardiomyopathy and the underlying mechanisms. Cardiac function of IL-6 knockout mice was significantly improved and interstitial fibrosis was apparently alleviated in comparison with wildtype (WT) diabetic mice induced by streptozotocin (STZ). Treatment with IL-6 significantly promoted the proliferation and collagen production of cultured cardiac fibroblasts (CFs). High glucose treatment increased collagen production, which were mitigated in CFs from IL-6 KO mice. Moreover, IL-6 knockout alleviated the up-regulation of TGFβ1 in diabetic hearts of mice and cultured CFs treated with high glucose or IL-6. Furthermore, the expression of miR-29 reduced upon IL-6 treatment, while increased in IL-6 KO hearts. Overexpression of miR-29 blocked the pro-fibrotic effects of IL-6 on cultured CFs. In summary, deletion of IL-6 is able to mitigate myocardial fibrosis and improve cardiac function of diabetic mice. The mechanism involves the regulation of IL-6 on TGFβ1 and miR-29 pathway. This study indicates the therapeutic potential of IL-6 suppression on diabetic cardiomyopathy disease associated with fibrosis.

Diabetic cardiomyopathy (DCM) is a distinct primary disease process that develops secondary to a metabolic insult in diabetic patients. DCM represents one of the major cardiovascular complications in diabetic patients and manifests by impaired diastolic and systolic cardiac function that may lead to heart failure^1^,^2^. Several mechanisms have been implicated in the pathogenesis of DCM. Among them, cardiac interstitial fibrosis has been shown to be the major pathological change, which worsens cardiac function by increasing the stiffness and reducing the compliance of heart wall due to excess collagen deposition^3^. The beneficial effects of several molecules on DCM have been attributed to the alleviation of cardiac interstitial fibrosis^4^,^5^.

Myocardial fibrosis is formed mainly due to the excessive proliferation of cardiac fibroblasts and the increased collagen synthesis and deposition. Inflammatory cytokines have been shown to be critical factors in regulating fibroblast proliferation, collagen secretion and interstitial fibrosis^6^. Interleukin 6 (IL-6) is one of the well-established cytokines involving in cardiac fibrosis. Sarkar S *et al.* observed that IL-6 treatment up-regulated collagen transcripts in cultured cardiac fibroblasts^7^. Meléndez GC *et al.* demonstrated that IL-6 treatment increased collagen production in cultured cardiac fibroblasts and promoted interstitial fibrosis of rat heart *in vivo*^8^. Remarkably, disruption of the expression of IL-6 was shown to be beneficial against fibrotic heart disease. González GE *et al.* showed that deletion of IL-6 prevented the development of cardiac dysfunction, myocardial inflammation, and fibrosis without altering the development of Ang II-high salt-induced hypertension and cardiac hypertrophy^9^. However, the role of IL-6 in DCM remains unknown.

MicroRNAs (miRNAs) are a class of single-stranded non-coding RNAs (20–22 nucleotides). An increasing body of evidence indicated that miRNAs extensively participate in the development of various heart diseases, including myocardial fibrosis^10^,^11^. Interestingly, in the cardiac tissue of streptozotocin (STZ) induced diabetic mice the expression of certain fibrotic-related microRNAs such as miR-21^12^, miR-24^13^, and miR-29^14^ were altered, implying that miRNAs may be involved in the pathogenesis of DCM by affecting fibrosis. Therefore, we hypothesized that IL-6 may regulate interstitial fibrosis of diabetic hearts by changing the expression of certain fibrotic-related miRNAs. In this study, we found that knockout of IL-6 alleviated the interstitial fibrosis and improved cardiac function in STZ induced diabetic mice. Furthermore, we confirmed that IL-6 regulates the proliferation and collagen synthesis by inhibiting TGFβ1 and miR-29 pathways.

## Results

### Levels of IL-6 in the serum and heart of mice with diabetic cardiomyopathy

**Figure 1:**
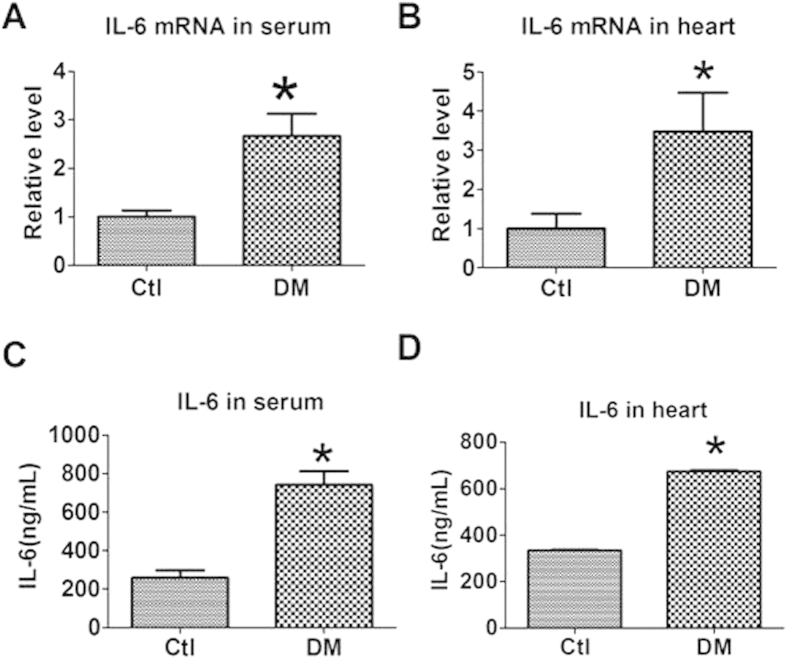
Level of IL-6 in the serum and heart of mice with diabetes mellitus (DM). (**A**,**B**) IL-6 mRNA level in the serum and heart by qRT-PCR. (**C**,**D**) IL-6 level in the serum and heart by Immunoassay. Data are expressed as mean ± SEM. N = 3. Ctl, control. ^*^P < 0.05 vs Ctl.

To evaluate the influence of diabetes mellitus on IL-6 expression, we established an STZ induced diabetic cardiomyopathy model in mice and detected the expression of IL-6 in both the serum and cardiac tissue 12-week’s after DM induction. We found that the mRNA level of IL-6 was significantly increased in both the serum and heart of diabetic mice than that of normal controls (Fig. 1A,B). Consistently, the protein level of IL-6 determined by immunoassay was also increased in both the serum and heart of diabetic mice (Fig. 1C,D).

### Effects of IL-6 knockout on cardiac dysfunction and interstitial fibrosis of diabetic mice

**Figure 2:**
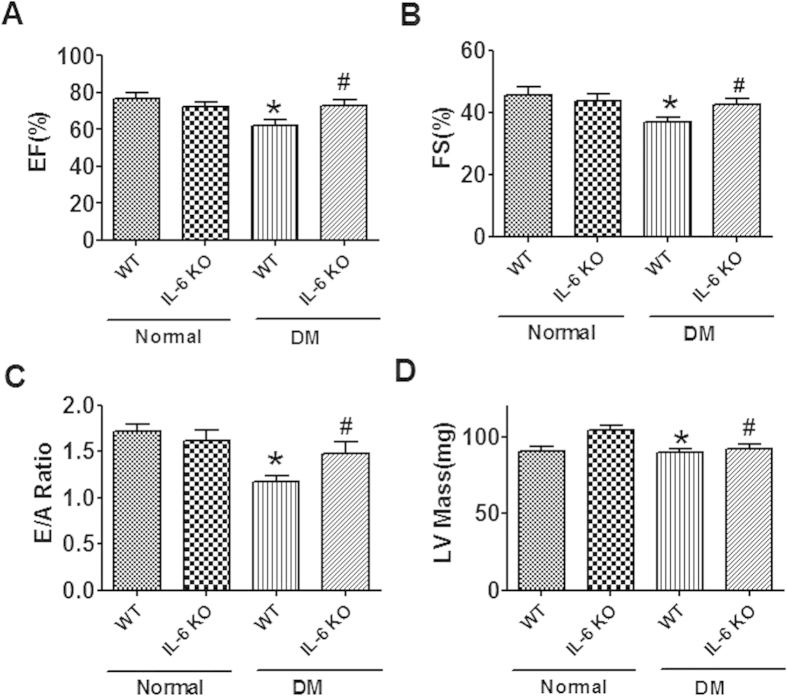
Effect of IL-6 knockout on cardiac function of diabetic mice by echocardiography. (**A**) Eject fraction (EF). (**B**) Fractional shortening (FS). (**C**) E/A ratio. (**D**) Left ventricular mass (LV Mass). WT, wild-type; IL-6 KO, IL-6 knockout; DM, Diabetes mellitus. Data are expressed as mean ± SEM. N = 7 or 8. *P < 0.05 vs normal WT, ^#^P < 0.05 vs DM WT.

We then explored the potential role of IL-6 in the pathogenesis of cardiac myopathy by employing IL-6 knockout mice. The blood glucose and organ parameters of mice were summarized in Table 1. Twelve weeks after DM induction, cardiac function was evaluated by echocardiography. We found that EF, FS and E/A ratio were all significantly decreased in the wild-type DM mice than wild-type controls, indicating the impairment of cardiac function (Fig. 2A–C). IL-6 knockout alleviated the deterioration of cardiac function induced by DM. The values of EF, FS and E/A ratio were all significantly higher in IL-6 knockout than WT mice after DM induction (Fig. 2A–C). The other cardiac dimensional parameters measured by echocardiography were shown in Table 2.

**Figure 3:**
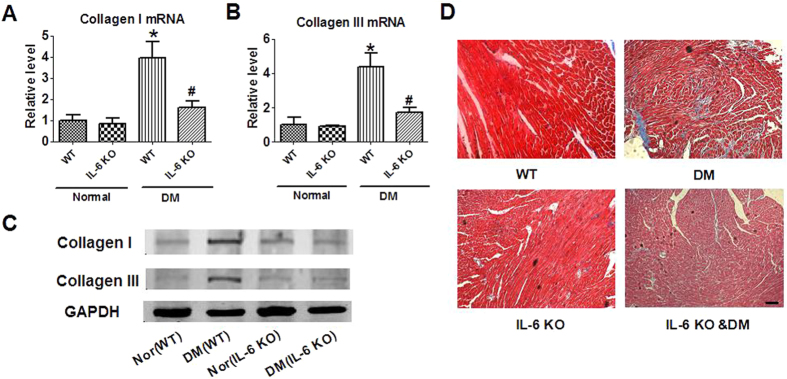
Effect of IL-6 knockout on interstitial fibrosis of diabetic mice. (**A**,**B**) mRNA levels of collagen I and collagen III. WT, wild-type; IL-6 KO, IL-6 knockout; DM, Diabetes mellitus. Data are expressed as mean ± SEM. n = 5. ^*^P < 0.05 vs normal WT, ^#^P < 0.05 vs DM WT. (**C**) Protein expression of collagen I and collagen III by western blot. WT, wild-type; IL-6 KO, IL-6 knockout; Nor, Normal; DM, Diabetes mellitus. (**D**) Deposition of collagen in cardiac tissue by Masson’s staining. Scale bar = 100 μm.

Interstitial fibrosis is a key pathological change that contributes to the impairment of cardiac function during various cardiac diseases. In this study, we found that the increased mRNA and protein expression of collagen I and collagen III after DM induction in the hearts of WT mice were reduced in the heart of diabetic IL-6 KO mice (Fig. 3A–C). Moreover, Masson’s staining showed less fibrotic area in the heart of diabetic IL-6 knockout mice than that of wild type mice (Fig. 3D). These data indicated that IL-6 is involved in the development of diabetic cardiomyopathy, and deletion of IL-6 is beneficial.

### Effects of IL-6 on the proliferation and collagen production of cultured cardiac fibroblasts (CFs)

**Figure 4:**
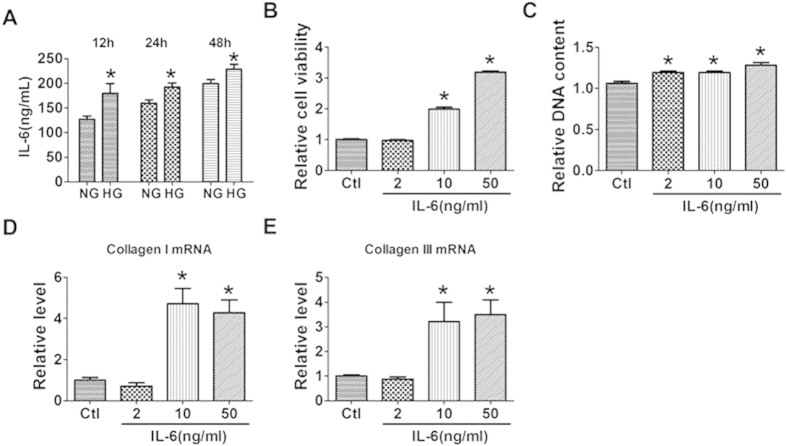
Effects of interleukin-6 (IL-6) on the proliferation of cultured cardiac fibroblasts (CFs). (**A**) Effects of glucose treatment on the production of IL-6 in CFs by immunoassay. (**B**,**C**) Effects of IL-6 treatment on the viability and proliferation of CFs by MTT assay and DNA incorporation assay. (**D**,**E**) mRNA levels of collagen I and collagen III in CFs treated with IL-6. Ctl, Control. NG, normal glucose; HG, high glucose. Ctl, control. Data are expressed as mean ± SEM. N = 4. *P < 0.05 vs NG or Ctl.

To further explore the role of IL-6 in interstitial fibrosis of diabetic cardiomyopathy, we firstly examined the influence of high glucose on the production of IL-6. We found that treatment with high glucose (HG, 25 mM) of cultured CFs significantly increased the production of IL-6 compared with that of normal glucose (NG, 5.5 mM) group (Fig. 4A). We then treated CFs with IL-6 to examine its direct effect on the proliferation and collagen production of CFs. We found that IL-6 treatment promoted the viability and proliferation of cultured CFs (Fig. 4B,C), and robustly increased the production of collagen I and collagenIII at the doses of 10 mg/mL and 50 ng/mL (Fig. 4D,E). These data indicated that HG induced upregualtion of IL-6 is attributable to the excessive production of collagen.

**Figure 5:**
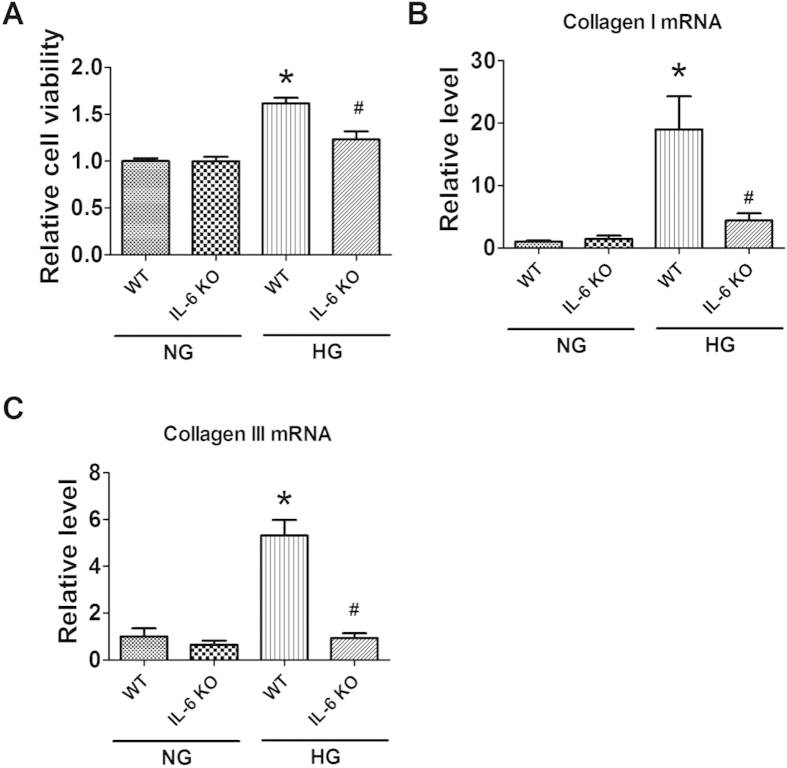
Effects of IL-6 knockout (IL-6 KO) on the proliferation of cultured cardiac fibroblasts (CFs) stimulated with high glucose (HG). (**A**) Cell viability evaluated by MTT assay. (**B**,**C**) mRNA levels of collagen I and collagen III. NG, Normal glucose. WT, Wild-type. Data are expressed as mean ± SEM. N = 4. *P < 0.05 vs NG WT, ^#^P < 0.05 vs HG WT.

We then investigate the influence of IL-6 knockout on the proliferation and collagen production in cultured CFs treated with HG. We found that HG treatment significantly increased cell viability and the production of collagen I and collagen III in cultured CFs from WT mice, which were alleviated in CFs from IL-6 KO mice (Fig. 5A–C). These data indicated that HG is able to induce the production of IL-6, which is attributable to the excessive production of collagen in CFs.

### Effects of IL-6 on the expression of TGFβ1 production

**Figure 6:**
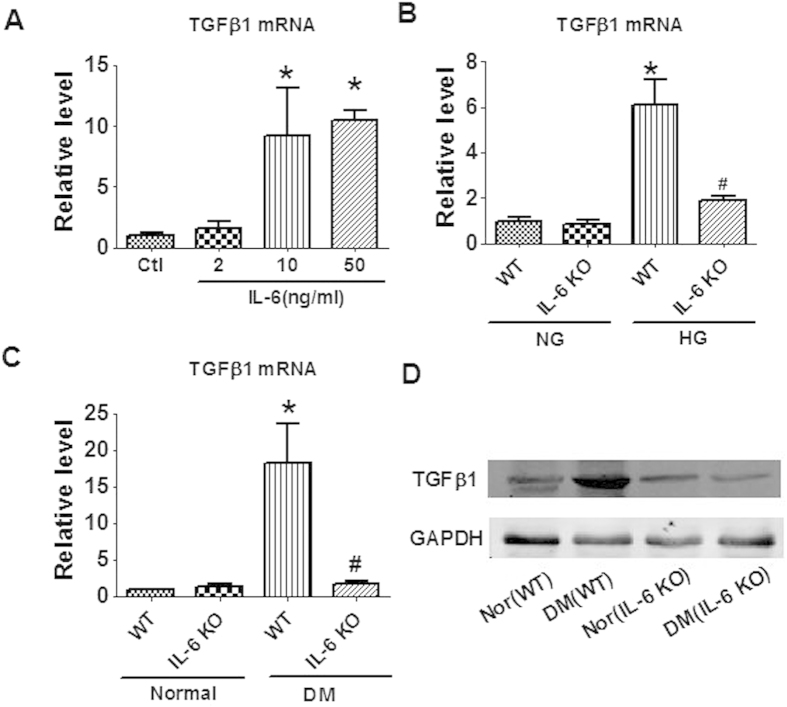
Effects of IL-6 on TGFβ1 production. (**A**) Application of IL-6 increased the production of TGFβ1 in cultured cardiac fibroblasts (CFs) from wild type (WT) mice. (**B**) Knockout of IL-6 inhibited the production of TGFβ1 induced by high glucose (HG) in cultured CFs. (**C**) Knockout of IL-6 inhibited the production of TGFβ1 in the heart of diabetic mice. (**D**) Protein expression of TGFβ1 by western blot. Ctl, control; NG, Normal glucose; Nor, Normal; DM, Diabetes mellitus; WT, Wild-type. Data are expressed as mean ± SEM. N = 4. *P < 0.05 vs WT or Ctl; ^#^P < 0.05 vs WT (HG or DM).

To explore the mechanism of IL-6′s regulation on fibrosis of diabetic heart, we examined the effect of IL-6 on TGFβ1, a key regulator of fibrosis. In cultured CFs, application of IL-6 increased the mRNA level of TGFβ1 (Fig. 6A). HG treatment also elevated the expression of TGFβ1 in WT CFs, which were alleviated in IL-6 KO CFs (Fig. 6B). Similarly, in diabetic hearts the upregulation of TGFβ1 at both mRNA and protein level is significantly suppressed by the deletion of IL-6 (Fig. 6C,D). These data imply that TGFβ1 is a mediator of the profibrotic action of IL-6 in diabetic cardiomyopathy.

### Role of miR-29 in mediating the regulation of IL-6 on CFs

**Figure 7:**
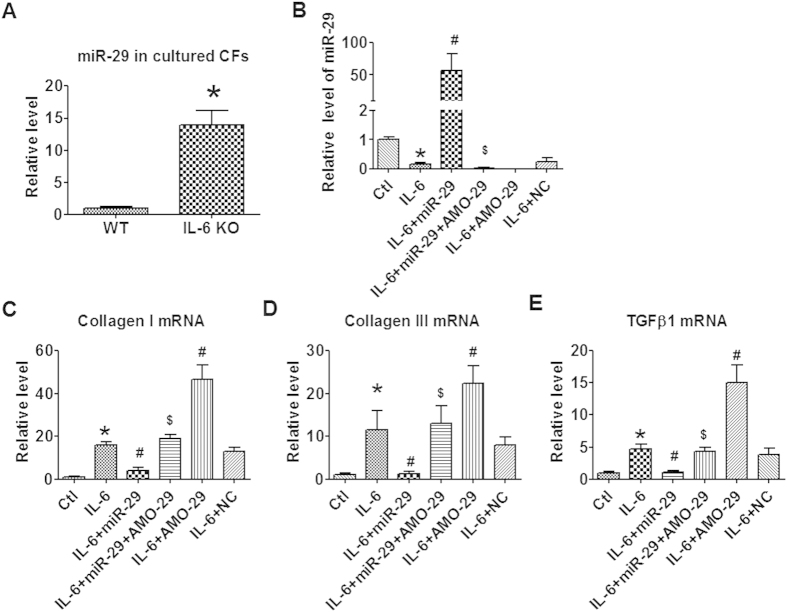
miR-29 on IL-6 induced collagenI and TGFB1 production in cultured CFs. (**A**) miR-29 levels in cultured cardiac fibroblasts (CFs) isolated from wild type (WT) and IL-6 knockout (IL-6 KO) mice. (**B**) miR-29 levels after IL-6 stimulation and exogenous miR-29/AMO-29 treatment. (**C**–**E**) mRNA levels of collagen I, collagen III and TGFβ1 in cultured CFs treated with IL-6 and miR-29/AMO-29. Ctl, Control; NC, Negative control. Data are expressed as mean ± SEM. N = 4. *P < 0.05 vs WT or Ctl; ^#^P < 0.05 vs IL-6; ^$^P < 0.05 vs IL-6 + miR-29.

miR-29 has been shown to be a critical regulator of cardiac fibrosis. We firstly tested the influence of IL-6 on miR-29 expression. We found that the level of miR-29 were significantly upregulated in cultured CFs of IL-6 KO mice that WT controls (Fig. 7A). In contrast, treatment of cultured CFs with IL-6 significantly reduced the level of miR-29 (Fig. 7B). These data indicated that the expression of miR-29 in CFs is regulated by IL-6. We then explored the regulatory role of miR-29 in IL-6 induced proliferation and collagen production of CFs. We found that forced overexpression of miR-29 abrogated IL-6 induced collagen I and III production, while knockdown of miR-29 expression with AMO-29 did the opposite (Fig. 7C,D). The effects of miR-29 on CFs were canceled by co-administration of AMO-29 (Fig. 7C,D). miR-29 also regulates the expression of TGFβ1 (Fig. 7E). The successful overexpression and knockdown of miR-29 were confirmed by qRT-PCR (Fig. 7B). These data indicated that inhibition of miR-29 also contributes to the profibrotic effects of IL-6 on CFs.

### Up-regulation of miR-29 in diabetic heart is compromised by IL-6 increment

**Figure 8:**
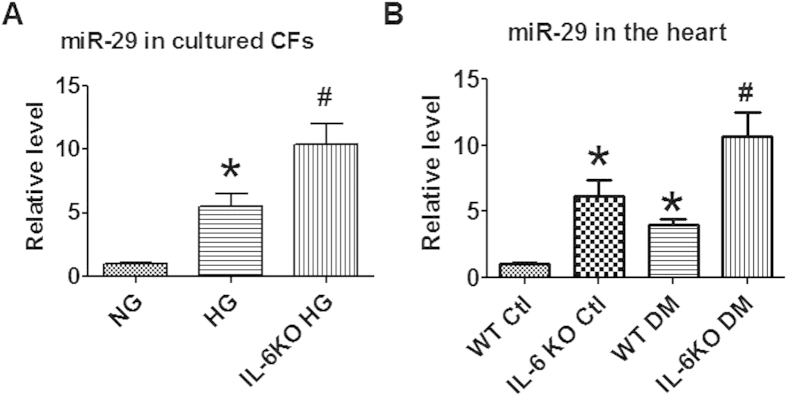
Effect of high glucose and IL-6 knockout on the expression of miR-29 in cultured CFs (**A**) and cardiac tissue (**B**) Data are expressed as mean ± SEM. N = 4. *P < 0.05 vs NG or WT; ^#^P < 0.05 vs HG or DM.

We evaluated the effects of high glucose on the expression of miR-29. The data showed that high glucose treatment increased the level of miR-29, which was further up-regulated when IL-6 was deleted (Fig. 8A). In diabetic mice, we found the same qualitative alteration. miR-29 increased in diabetic hearts than controls (Fig. 8B). Interestingly, in IL-6 knockout mice the level of miR-29 in the heart further elevated than WT controls upon diabetes induction (Fig. 8B). These data implied that upregulation of miR-29 may be protective response of the hearts to diabetic stimuli. Suppression of miR-29 upregulation may be one of the mechanisms responsible for the deleterious role of IL-6 in diabetic cardiomyopathy.

## Discussion

In this study we demonstrated that IL-6 knockout is protective against DCM. IL-6 knockout improved cardiac function and alleviated interstitial fibrosis in STZ induced diabetic mice. IL-6 application promoted the proliferation and collagen production of CFs, while knockout of IL-6 mitigated HG stimulated collagen production of CFs. Furthermore, we confirmed that IL-6 participates in the regulation of HG induced collagen production of CFs by enhancing the expression of TGFβ1 and inhibiting the expression of miR-29.

IL-6 is a pro-inflammatory cytokine that is upregulated in various cardiac diseases. IL-6 is elevated in both the plasma and cardiac tissue of myocardial infarction rats^15^. In patients with peripartum cardiomyopathy, the level of IL-6 is higher than that of controls^16^. Higher plasma levels of interleukin-6 were observed in patients with diastolic heart failure than the control subjects^17^. IL-6 was also shown to be closely related to diabetes. Pickup *et al.* showed that serum IL-6 concentration was higher in type 2 Diabets Mellitus (T2DM) patients than non-diabetic subjects^18^. Moreover, the serum IL-6 level of T2DM patients with more than two features of the metabolic syndrome was higher than those with less than two features of the metabolic syndrome^18^. Consistently, in this study we found that IL-6 level were both increased in the serum and cardiac tissue of diabetic mice than non-diabetic controls.

Not just act as a clinical marker, IL-6 has been shown to be deeply involved in the pathogenesis of cardiac diseases and cardiovascular complications of diabetes. Deletion of IL-6 inhibited cardiac inflammation, fibrosis and dysfunction in an angiotensin II and high salt induced hypertension model of mice^9^. Antibody against IL-6 ameliorated left ventricular remodeling of chronic infarct mice^19^. In contrast, IL-6 signaling seems to be protective in acute cardiac diseases. Loss of IL-6 disrupted exercise preconditioning against myocardial ischemia reperfusion injury^19^. IL-6 pretreatment induces a delayed cytoprotective response in cultured cardiac myocytes via activating of phosphatidylinositol (PI) 3-kinase/Akt pathway and promoting nitric oxide production^20^. Similarly, in the IL-6 knockout mouse model, the late preconditioning mediated cardiac protection was abolished^21^. However, the role of IL-9 in diabetic cardiomyopathy remains unclear. In this study, we for the first time evaluated the role of IL-6 in cardiac remodeling 12 weeks after diabetes induction in mice. In consistent with observations in previous chronic heart diseases^9^,^19^, we found that IL-6 is pathogenic in diabetic cardiomyopathy as deletion of IL-6 alleviated the impairment of cardiac function and interstitial fibrosis of diabetic hearts. On the contrary, Fuchs M *et al.* demonstrated that IL-6 deletion produced no effects on the adaptation of the left ventricle to myocardial infarction in the long term^22^. One explanation for the discrepancy may be the different pathological processes of chronic cardiac infarction and diabetic cardiomyopathy.

Interstitial fibrosis is the critical pathological change of DCM, which will increase the stiffness of ventricular wall and reduced the compliance of the heart that finally leads to cardiac dysfunction. IL-6 is a key cytokine in regulating cardiac fibrosis. Application of IL-6 promoted the proliferation and collagen production of CFs and interstitial fibrosis^7^,^8^. Conversely, suppression of IL-6 signaling inhibited cardiac interstitial fibrosis^9^. Considering the fact that IL-6 is elevated during DCM, it is reasonable to speculate that IL-6 up-regulation underlies the interstitial fibrosis of DCM. In this study, we confirmed that IL-6 is secreted by CFs and treatment with high glucose stimulated the secretion of IL-6. Moreover, knockout of IL-6 inhibited the proliferation and collagen production of cultured CFs. These data provided direct evidence on the causal role of IL-6 in high glucose induced fibrosis.

**Figure 9:**
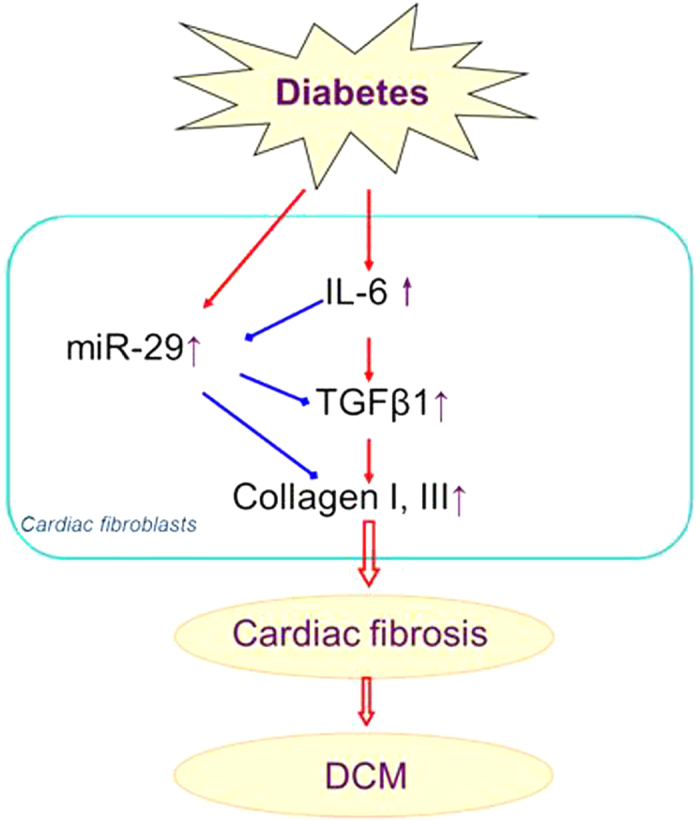
A schematic graph on the signaling pathway of the regulation of IL-6 on cardiac fibrosis and DCM.DCM, diabetic cardiomyopathy. Blue line with hammer head for “inhibition”; red line with arrow head for “stimulation”.

TGFβ1 is the critical regulator of fibrosis-related signaling pathway. In the present study we found that TGFβ1 is upregulated by application of IL-6 and suppressed upon IL-6 deletion, indicating that TGFβ1 pathway is involved in the pro-fibrotic effects of IL-6. MiRNAs were shown to be deeply involved in cardiac fibrosis^12^,^23^. Van Rooij E *et al.* demonstrated that miR-29 produced anti-fibrotic effect by directly inhibiting the expression of collagen I and III^13^. Moreover, a recent study demonstrated that miR-29 inhibited angiotensin II-induced cardiac fibrosis by targeting TGF-β/Smad3 signaling^24^. However, the role of miR-29 in IL-6 induced collagen production and diabetic cardiomyopathy remains unknown. In this study, we for the first time evaluated the involvement of miR-29 in interstitial fibrosis of diabetic cardiomyopathy. In consistent, we found that overexpression of miR-29 cancelled the pro-fibrotic effects of IL-6 in cultured CFs and inhibited the expression of TGFβ1. These findings imply that IL-6 regulates interstitial fibrosis of DCM through miR-29/TGFβ1 pathway. Interestingly, studies showed that miR-29 is also negatively regulated by TGFβ1 signaling pathway in renal fibrosis^25^ and mouse myoblasts^26^. These data imply that there may be a negatively reciprocal regulatory loop existing between miR-29 and TGFβ1, which requires further study. Moreover, we found the level of miR-29 increased in diabetic hearts (Fig. 8), which is in consistent with previous studies^27^,^28^. Interestingly, knockout of IL-6 further elevated the level of miR-29 than WT diabetic hearts (Fig. 8). These data indicated that upregulation of miR-29 represents a protective response of the hearts to diabetic stimuli, which were compromised by the production of IL-6. The potential signaling pathway of IL-6 in DCM was summarized in Fig. 9.

In conclusion, we demonstrated that IL-6 deletion is beneficial against DCM by alleviating interstitial fibrosis. IL-6 modulates the development of high glucose induced cardiac fibrosis by affecting TGFβ1 and miR-29 pathways.

## Materials and Methods

### Animals

Male C57BL/6 wild-type (WT) mice were purchased from the Animal Center of the Second Affiliated Hospital of Harbin Medical University (Harbin, China). IL-6 knockout mice was graciously provided by Prof. Zhinan Yin (Tianjin University, China). Mice were kept under standard animal room conditions (Temperature, 21 ± 1 °C; Humidity, 55–60%) with food and water ad libitum. All animal studies were approved by the Institutional Animal Care and Use Committee of Harbin Medical University, P.R. China. All animal care and experimental procedures were in accordance with the regulations of the Institutional Animal Care and Use Committee of Harbin Medical University. Mice (8-week old) received three consecutive intravenous injections of STZ (50 mg/kg, Sigma, St. Louis, MO) in citrate buffer (pH 4.6). For vehicle control mice, only citrate buffer was administered. Plasma glucose levels were measured at the beginning and 12 weeks after STZ injection by using Contour glucose meter (Roche, Germany). Mice with fasting plasma glucose over 11.1 mM were considered diabetic. Twelve weeks after diabetes induction, the diabetic mice were subjected to subsequent experiments.

### Cardiac echocardiography

Mice were anesthetized with a cocktail of ketamine, xylazine, and atropine (100 mg/kg, 10 mg/kg, and 1.2 mg/kg, respectively, i.p.) for echocardiography. Both two-dimensional M-mode and three-dimensional Doppler echocardiography were performed by using the Vevo 770 imaging system (VisualSonics, Toronto, Canada) to evaluate cardiac diameter and function. Derived echocardiography parameters included LV ejection fraction (LVEF) and LV fractional shortening (LVFS). The ratio of early to late mitral inflow velocity (E/A) and E velocity deceleration time were used to assess LV filling, which were obtained by the apical four-chamber view at the level of mitral inflow.

### Cardiac fibroblasts (CFs) isolation and culture from neonatal mice

Neonatal mouse cardiac fibroblasts were collected in accordance with the following procedure: twenty or more hearts from 3-day old C57BL/6 mice were cut and placed together in 0.25% trypsin. Pooled cell suspensions were centrifuged and resuspended in Dulbecco’s modified Eagle’s medium (DMEM Hyclone, USA) supplemented with 10% fetal bovine serum, 100U/ml penicillin and 100 μg/ml streptomycin. The suspension was incubated in culture flasks for 90 minutes, which makes fibroblasts preferentially adhere to the bottom of the culture flasks. Non-adherent and weakly attached cells were removed and the medium was changed. Cell cultures were incubated at 37 °C in a humidified atmosphere of 5% CO_2_ and 95% air. Both high glucose (25 mM) and normal glucose (5.5 mM) mediums were used in culturing CFs.

### Cell Culture and Transfection

Cardiac fibroblasts were cultured in DMEM (Hyclone, USA) supplemented with 10% fetal bovine serum. When the cells grew to 60% confluence, they were treated with IL-6 cytokines (50 pg/ml) and transfected with miR-29 mimics (miR-29), miR-29 inhibitor (AMO29) or negative control (Scramble) using X-tremeGENE siRNA Transfection Reagent (Roche, Germany). After 48 hours, cells were collected and applied to other experiments.

### Cytokine Assays

Mice were sacrificed 12 weeks after the establishment of diabetes model. Blood sample and cardiac tissue were collected and IL-6 levels was measured using enzyme-linked immunosorbent assay kits (RayBiotech. Inc. Norcross GA, USA). Supernatants from cultured cardiac fibroblasts were also collected for IL-6 assay.

### Proliferation Assay of CFs

MTT and CyQUANT® NF assay were used to measure cardiac fibroblasts proliferation. CFs were seeded onto 96-well flask culture (5 × 103 cells per well) in RPMI-1640 (Hyclone, USA) supplemented with 10% fetal bovine serum. After culturing for 24 hours, IL-6 cytokines (50 pg/ml; Sigma) alone or in combination with miR-29, miR-29 + AMO-29, or negative control were added to the medium. After 48-hour’s incubation, the CFs were treated with MTT (5 mg/mL) for 4 hours at 37 °C. The CyQUANT® NF assay was performed according to the manufacturer’s instructions. A microplate reader was used to measure the final MTT and CyQUANT® NF assay samples.

### Total RNA extraction and real-time RT-PCR

Total RNA was extracted from serum, fibroblasts, and cardiac tissues by using Trizol reagent (Invitrogen, USA) according to manufacturer’s protocol. Total RNA (0.5 μg) was reversed by using the TransScript reverse transcriptase (GMO technology, Beijing) to obtain cDNA. The RNA levels of collagen I, collagen III, TGF-β1 and IL-6 were determined using SYBR Green I incorporation method on ABI 7500 fast Real Time PCR system (Applied Biosystems,USA), with GAPDH as an internal control for mRNAs and U6 for miR-29. The primers are:

miR-29a F: 5′-CCGTAGCACCATCTGAAATC-3′

miR-29a R: 5′-GTATCCAGTGCGTGTCGTG-3′

U6 F: 5′- GCTTCGGCAGCACATATACTAAAAT -3′

U6 R: 5′- CGCTTCACGAATTTGCGTGTCAT -3′

Collagen I F: 5′- AAGAAGACATCCCTGAAGTCA -3′

Collagen I R: 5′- TTGTGGCAGATACAGATCAAG -3′

Collagen III F: 5′- TTGGGATGCAGCCACCTTG -3′

Collagen III R: 5′- CGCAAAGGACAGATCCTGAG -3′

TGF-β1 F: 5′- GTGTGGAGCAACATGTGGAACTCTA -3′

TGF-β1 R: 5′- TTGGTTCAGCCACTGCCGTA -3′

IL-6 F: 5′- GTGTGGAGCAACATGTGGAACTCTA -3′

IL-6 R: 5′- TTGGTTCAGCCACTGCCGTA -3′

GAPDH F: 5′- AAGAAGGTGGTGAAGCAGGC-3′

GAPDH R: 5′- TCCACCACCCTGTTGCTGTA-3′

### Western blot

The total protein (60 μg) extracted from cardiac tissues was fractionated by SDS-PAGE (10% polyacrylamide gels) and transferred to nitrocellulose membrane. The membrane was blocked with 5% nonfat milk for 1.5 h at room temperature. The membrane was then incubated with primary antibodies collagen I (1:800 dilution, Proteintech, China), collagen III (1:2000 dilution, Proteintech, China), TGFβ1 (1:200 dilution, Proteintech, China) and GAPDH (1:1000 dilution, Kangcheng Inc, China) on shaking bed overnight at 4 °C. The membrane was washed with PBS-T for 3 times and incubated with secondary antibodies for 1 h at room temperature. Finally, the membranes were rinsed with PBS-T before scanned by Imaging System (LI-COR Biosciences, Lincoln, NE, USA).

### Masson staining

The left ventricle fixed in 4% paraformaldehyde were embedded with paraffin, and cross-sectionally cut into 5 μm thick sections. Masson’s trichrome staining was used to evaluate collagen deposition. Sections were imaged at 200× magnification by bright-field microscopy (IX71 Olympus, Japan).

### Synthesis and transfection of miR-29 and anti-miR-29 antisense inhibitor

miR-29a and its inhibitor anti-miR-29 (AMO-29) were synthesized by Genepharma (Shanghai). The sequence of miR-29a is: 5′-UAGCACCAUCUGAAAUCGGUUA-3′. The sequence ofAMO-29a is 5′-UAACCGAUUUCAGAUGGUGCUA-3′. miRNAs were transfected to cultured CFs using X-tremeGENE siRNA Transfection Reagent (Roche, Germany).

### Statistical analysis

All data were expressed as mean ± SEM. Statistical analysis was performed using Student’s non-paired t test or Kruskal-Wallis H test. Differences were considered as statistically significant when P < 0.05.

## Additional Information

**How to cite this article**: Zhang, Y. *et al.* Deletion of interleukin-6 alleviated interstitial fibrosis in streptozotocin-induced diabetic cardiomyopathy of mice through affecting TGFβ1 and miR-29 pathways. *Sci. Rep.* **6**, 23010; doi: 10.1038/srep23010 (2016).

## Figures and Tables

**Table 1:** Systemic and organ parameters of mice.

	WT Sham (n = 8)	WT DM (n = 8)	IL-6KO Sham (n = 8)	IL-6KO DM (n = 8)
Blood glucose (mM)	6.16 ± 0.19	29.6 ± 0.32^**^	5.80 ± 0.21	27.9 ± 0.21^##^
BW (g)	32.8 ± 0.48	24.5 ± 0.31	30.5 ± 0.59	27.8 ± 0.45
HW (mg)	160 ± 4.2	140 ± 3.8	140 ± 6.1	130 ± 5.7
LW (mg)	150 ± 3	140 ± 4.6	130 ± 3.6	150 ± 9.7
HW/BW (mg/g)	4.77 ± 0.11	5.88 ± 0.13^**^	4.50 ± 0.19	4.78 ± 0.17^@@^
LW/BW (mg/g)	4.55 ± 0.10	5.59 ± 0.24	4.37 ± 0.15	5.26 ± 0.37

BW, body weight; HW, heart weight; LW, lung weight. Data are expressed as mean ± SEM. ^**^P < 0.01 vs WT Sham; ^##^P < 0.01 vs IL-6KO Sham; ^@@^P < 0.01 vs WT DM.

**Table 2:** Cardiac dimensional parameters measured by echocardiography.

	WT Sham (n = 8)	WT DM (n = 8)	IL-6KO Sham (n = 8)	IL-6KO DM (n = 8)
IVSd (mm)	0.82 ± 0.06	1.10 ± 0.05**	0.72 ± 0.04	0.95 ± 0.05
IVSs (mm)	1.22 ± 0.09	1.59 ± 0.07**	1.14 ± 0.06	1.22 ± 0.07##
LVIDd (mm)	2.61 ± 0.18	3.08 ± 0.20	2.64 ± 0.16	2.87 ± 0.16
LVIDs (mm)	1.42 ± 0.12	1.99 ± 0.11**	1.54 ± 0.07	1.73 ± 0.13
LVPWd (mm)	0.92 ± 0.07	1.20 ± 0.09*	0.90 ± 0.04	0.90 ± 0.07#
LVPWs (mm)	1.27 ± 0.10	1.59 ± 0.09*	1.10 ± 0.06	1.20 ± 0.05##

IVSd, diastolic interventricular septum diameter; IVSs, systolic interventricular septum diameter; LVIDd, diastolic left ventricular internal diameter; LVIDs, systolic left ventricular internal diameter; LVPWd, diastolic left ventricular posterior wall diameter; LVPWs, systolic left ventricular posterior wall diameter; Data are expressed as mean ± SEM. *P < 0.05, **P < 0.01 vs WT Sham; ^#^P < 0.05, ^##^P < 0.01 vs WT DM.
